# Chromosomal markers in the genus *Karenia*: Towards an understanding of the evolution of the chromosomes, life cycle patterns and phylogenetic relationships in dinoflagellates

**DOI:** 10.1038/s41598-018-35785-7

**Published:** 2019-02-28

**Authors:** Ángeles Cuadrado, Alfredo De Bustos, Rosa I. Figueroa

**Affiliations:** 1 Universidad de Alcala (UAH), Dpto Biomedicina y Biotecnología, 28805 Alcalá de Henares, Madrid, Spain; 2 Instituto Español de Oceanografia (IEO), Subida a Radio Faro 50, 36390 Vigo, Spain; 3 Aquatic Ecology, Biology Building, Lund University, 22362 Lund, Sweden

## Abstract

Dinoflagellates are a group of protists whose genome is unique among eukaryotes in terms of base composition, chromosomal structure and gene expression. Even after decades of research, the structure and behavior of their amazing chromosomes—which without nucleosomes exist in a liquid crystalline state—are still poorly understood. We used flow cytometry and fluorescence *in situ* hybridization (FISH) to analyze the genome size of three species of the toxic dinoflagellate genus *Karenia* as well the organization and behavior of the chromosomes in different cell-cycle stages. FISH was also used to study the distribution patterns of ribosomal DNA (45S rDNA), telomeric and microsatellites repeats in order to develop chromosomal markers. The results revealed several novel and important features regarding dinoflagellate chromosomes during mitosis, including their telocentric behavior and radial arrangement along the nuclear envelope. Additionally, using the (AG)_10_ probe we identified an unusual chromosome in *K. selliformis* and especially in *K. mikimotoi* that is characterized by AG repeats along its entire length. This feature was employed to easily differentiate morphologically indistinguishable life-cycle stages. The evolutionary relationship between *Karenia* species is discussed with respect to differences in both DNA content and the chromosomal distribution patterns of the DNA sequences analyzed.

## Introduction

Dinoflagellates are a large and diverse group of protists widely distributed in marine and freshwater environments. Most are microalgae and have been extensively studied with respect to their capacity to form toxic and noxious blooms (commonly known as red tides). However, the dinoflagellate group is highly diverse and also includes heterotrophic species, saprophytes, parasites and essential symbionts of reef-building corals^1^. Moreover, dinoflagellates are exceptional among eukaryotes due to their atypical nuclei (see^2^ for a review) and the organization of their genome, which differs from that of other eukaryotes with respect to base composition and chromosomal organization (see^3^ for review). Yet, while dinoflagellates and their chromosomes have been extensively studied for decades, the organization and behavior of dinoflagellate chromosomes during mitosis and meiosis and during specific processes, such as replication and transcription, remain poorly characterized.

The genomes of some dinoflagellate species are among the largest in eukaryotes, ranging in size from 1.5 to 112 Gbp^4,5^. They are organized into hundreds of chromosomes, generally lack histones^6,7^ and therefore nucleosomal organization as well. Genomic sequence information for dinoflagellates is available but for free-living dinoflagellates the large size of their genomes has hindered efforts to obtain a sequenced reference genome and therefore a broader understanding of the organization and function of dinoflagellate genomes^8,9^. For example, it is still unclear whether the high DNA content is related to polyploidy. At the molecular level, the nuclear DNA of dinoflagellates is characterized by the presence of 5-hydroximethyluracil, which replaces up to 70% of thymidines in several species, a high G-C content and a high proportion of repeated DNA^10^. Although how dinoflagellates regulate transcription without histones is still unknown, it has been shown that active chromatin allows transcription to occur in peripheral DNA loops where supercoiled chromatin is locally untwisted^4^. In general, dinoflagellate genes are present in high copy number. Some of these genes are arranged in tandem^4^ and are transcribed on a single mRNA that possesses a splice leader sequence unique to all mRNAs^11^. Transcriptome analysis of different species has revealed the presence of eukaryotic genes that encode the four core nucleosomal histones (H2A, H2B, H3 and H4), in addition to several families of prokaryotic histone-like proteins^8^. Nuclear DNA is associated with basic nuclear proteins at a protein/DNA ratio of 1:10, in contrast to the 1:1 ratio found in essentially all other eukaryotes and the even lower ratio of prokaryotes. But how dinoflagellate chromosomes are organized into functional chromatin remains an enigma.

Another original feature of dinoflagellates is that their chromosomes remain condensed throughout the cell cycle and are thus permanently visible by light microscopy. On transmission electron microscopy (TEM), the chromosomes show a peculiar fibrillary and arched organization^3^ that corresponds to a cholesteric liquid crystal structure, evident as birefringence under polarized light^12^. In the absence of nucleosomes, chromatin organization may be mediated by high levels of divalent cations (Ca^2+^, Mg^2+^) and by transition metals, RNA, and low levels of highly basic nucleoproteins that bind DNA with high affinity^13^.

Most dinoflagellates have haplontic life cycles, in which the growth stage is haploid and the cells divide by mitosis (asexual reproduction) during the course of a eukaryotic-type cell cycle that includes G1, S, G2 and M phases as well as their specific check points^14^. Chromosomal condensation patterns vary during the cell cycle^15^, with maximum unwinding occurring in S phase^16^. However, dinoflagellate mitosis also shows distinctive features that have prevented a clear identification of the different mitotic phases, including the absence of a breakdown of the nuclear envelope (endomitosis) and nucleolar disassembly^17^. In addition, dinoflagellate mitosis is not characterized by a typical metaphase plate^16^ and the segregation of daughter chromatids attached to the nuclear envelope is mediated by a mitotic spindle that appears within cytoplasmic channels surrounded by the nuclear envelope and adjacent to the site of chromosomal attachment. TEM studies of chromosomes have revealed the presence of chromatids coiled together, except during division, when they split longitudinally into characteristic V or Y shapes^2,18^. Since centromeres or kinetochores have not been identified, how the daughter chromatids of dinoflagellates separate and move to opposite poles is unknown. Besides asexual reproduction, which allows rapid proliferation and the formation of dense blooms, sexual reproduction is essential for the adaptation and survival of dinoflagellates, since it promotes genetic recombination during meiosis. Indeed, sexual reproduction is key to explaining the recurrence of many blooms. During the sexual phase, two haploid cells (gametes) fuse to form a diploid mobile zygote (planozygote). In general, the most common pathway of sexual reproduction comprises planozygote encystment to yield a quiescent, benthic phase (hypnozygote) referred to as a resting cyst. Meiosis of the planozygote and/or hypnozygote restores the vegetative stage^19,20^. However, although a sexual life stage has been reported in many dinoflagellates species in stock cultures, it is poorly understood, in part because vegetative cells, gametes and planozygotes are morphologically very similar and therefore difficult to study individually. Sexuality studies in dinoflagellates are therefore mainly based on morphological features, evidenced by the presence of pairs of small cells (mating gametes) that in most cases are derived from normal sized vegetative cells, and the presence of resting cysts^20,21^.

By bringing together cytogenetics and molecular biology, fluorescence *in situ* hybridization (FISH) has enhanced the accuracy and efficiency of cytogenetic analysis. FISH can be used to investigate many features of nuclei and chromosomes, in both animals and plants. These capabilities explain the ever-growing popularity of the technique and its routine use in many laboratories. However, FISH-based approaches remain a challenge in dinoflagellates, given the unique nature and behavior of the nuclei and chromosomes of these organisms during mitosis. For example, the substantial amounts of 5’hydroxymethyluracil, the low melting temperature of the DNA and the highly condensed state of dinoflagellate DNA hinder the accessibility of probes targeting specific sequences^22^. Consequently, the use of FISH has thus far been limited to the identification of the telomeres of a few dinoflagellate species^23,24^ and to the localization of ribosomal DNA genes (rDNA) in species of the genus *Alexandrium*^25^.

The ultrastructure of dinoflagellate chromosomes has been examined by electronic microscopy in studies conducted over the last 35 years but direct light microscopy investigations of the morphology of “dinochromosomes” is scarce. The molecular organization of these nucleosome-less chromosomes is still poorly understood and the presence of constrictions and other structural characteristics, such as regions of eu-/heterochromatin, has yet to be adequately described.

The dinoflagellate genus *Karenia* includes 12 described species found throughout the world and mostly known by their ability to produce toxins than are lethal to fish and other marine organisms. The focus of this study was three closely related species, *K. brevis, K. mikimotoi*, and *K*. *selliformis*, that cause harmful algal blooms in different parts of the world but which also co-exist during *Karenia* blooms. Due to their harmful effects, these three species have been entered into the IEO-UNESCO Taxonomic Reference List of Harmful MicroAlgae (http://www.marinespecies.org/hab/index.php). *K. brevis* produces brevitoxins, which cause neurotoxic shellfish poisoning and respiratory stress in humans (see review by^26^), whereas *K. selliformis* and *K. mikimotoi* produce neurotoxic shellfish toxins^27^.

In this work we applied FISH and non-denaturing (ND)-FISH to analyze the chromosomal distribution of the 45S rDNA genes of these three *Karenia* species as well as the repeated sequences found in the telomeres and four microsatellites (AG, AC, GACA and GATA). Our aim was to provide new data on the morphology, organization and behavior of dinoflagellate chromosomes during both the cell cycle and the dinoflagellate life cycle. The physical location of these DNA sequences was used to investigate chromosomal organization with respect to genome structure and function. These chromosomal markers thus allowed both the three *Karenia* species and sexual vs. asexual life stages to be distinguished. The latter was particularly important since, morphologically, diploid cells (zygotes) of *Karenia* are very difficult to distinguish from vegetative (haploid) cells^28^. Furthermore, the development of these chromosomal markers provides a new tool with which to explore potential relationships between *Karenia* strains or species isolated from different geographical blooms. Previously, this was mainly done on a morphological basis, in some cases with the support of DNA sequence data^29,30^.

## Results

### DNA content, nuclear size and chromosome number

The DNA content of the three *Karenia* strains was calculated with respect to that of clonal strains of *Alexandrium minutum* with a known genome size. According to Stüken *et al*. (2015), the genome of *A. minutum* strain AMP4 is slightly larger than that of strain VGO 577 (Supplementary Figure S1A): 26.2 ± 3.0 vs. 25.7 ± 3.2 pg DNA/cell, respectively. The coefficients of variation (CVs) of the 1 C peaks of the *Karenia* strains were typically between 5 and 7. Among the species analyzed, *K. brevis* had the largest genome (196.7 ± 6.7 pg DNA/cell), followed by *K. selliformis* (158.6 ± 9.5 pg DNA/cell) and *K. mikimotoi* (53.4.1 ± 3.05 pg DNA/cell) (Fig. S1A,B).

**Figure 1:**
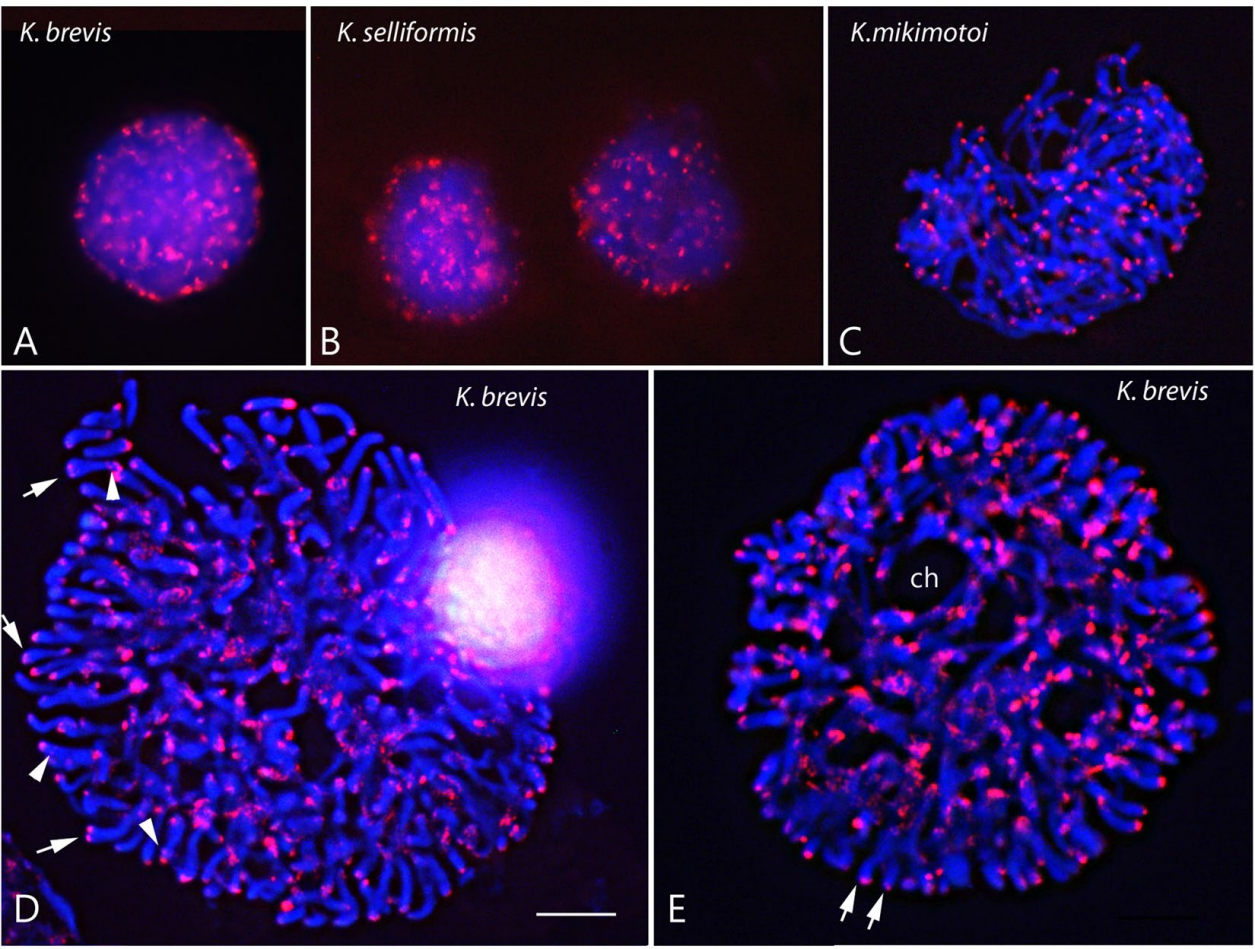
Physical mapping of the telomeric repeats in the nuclei of three *Karenia* species at different stages of the asexual cycle. *K. brevis* (**A**,**D**,**E**), *K. selliformis* (**B**) and *K. mikimotoi* (**C**). Each panel shows merged images, to facilitate visualization of the DAPI-stained DNA (blue) and *in situ* hybridization of the Dy547-labeled oligonucleotide (CCCTAAA)_3_ used to localize the telomeric repeats (red) during interphase (**A**,**B**) and mitotic stages (**C**–**E**). In D, the arrows indicate double visualized telomeric signals, and the arrowheads interstitial signals. The pairs of arrows in E indicate separate sister chromatids. ch = cytoplasmic channels. Scale bar = 10 μm for all panels.

**Figure 2:**
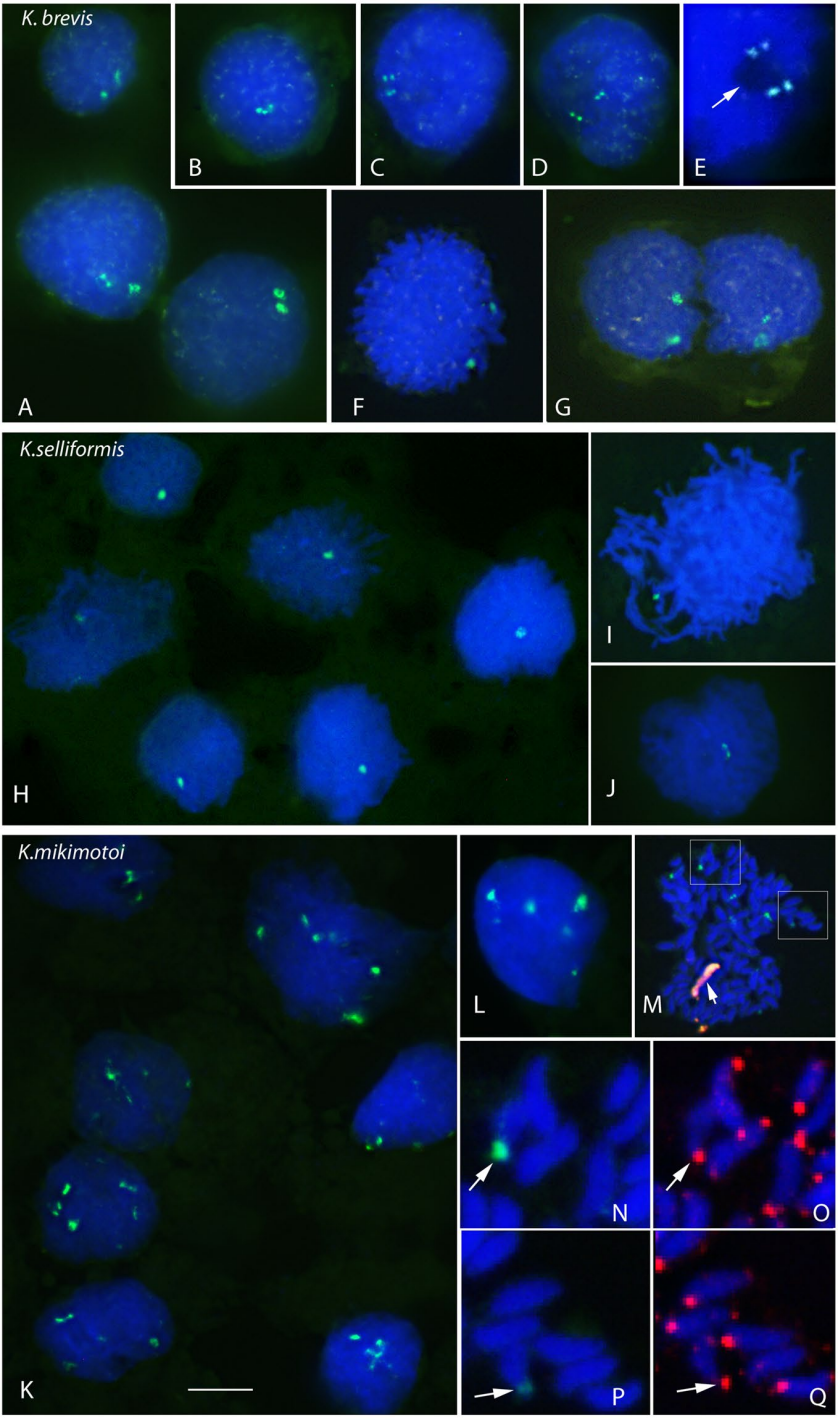
Physical mapping of 45S rDNA in three *Karenia* species. DAPI-stained DNA (blue) and *in situ* hybridization of the digoxigenin-labeled pTa71 probe for the detection of 45S rDNA (green) in *K. brevis* (**A–G**), *K. selliformis* (**H–J**) and *K. mikimotoi* (**K–Q**) nuclei at different stages of the vegetative cell cycle. In E, the arrow points to a nucleolus at higher magnification to show the co-localization of rDNA in this less-intense DAPI-staining nuclear domain. M–Q provide an example of two-color FISH using the 45S rDNA (green) and (AG)_10_ probes (red) before ND-FISH using the telomeric probe. In M, the arrow indicates the AG-chromosome, and the boxes the nuclear areas amplified in N, O and in P, Q to show the precise co-localization of the 45S rDNA (arrows in N, P) with the telomeric signals (arrows in O, Q). Scale bar = 10 μm (A–D, F–M) or 2.5 μm (E, N–Q).

The nuclei of unsynchronized cultures of the three species varied greatly in their appearance depending on the cell-cycle and life-cycle stage, but they were generally spherical, with well-defined chromosomes lying close to each other during both interphase and mitosis (Fig. 1). Although dinoflagellate chromosomes are, as noted above, permanently condensed throughout the cell cycle, chromosomes with different degrees of compaction were observed, clearly indicating a transient unwinding during genome replication in S phase, given that transcription occurs on DNA loops of locally decondensed chromatin. Based on the appearance of the nuclei and chromosomes, we were able to differentiate two main groups of cells. Interphasic nuclei (G1, S or G2) were those with a high degree of nuclear compaction, in which nuclear areas were smaller and the chromosomes close to each other (Fig. 1A,B). Mitotic nuclei were those with highly condensed but separated chromosomes, in which splits along the length of the chromosomes or cytoplasmic channels were observed (Fig. 1C–E). However, due to the presence of the nuclear envelope during the entire cell cycle, even in mitotic nuclei, it was very difficult to separate the chromosomes individually. This prevented us from precisely determining the number of chromosomes for the three species, even in nuclei with the maximum degree of chromosomal compaction (e.g., Fig. 2M). Moreover, the inexistence of a classical metaphase, during which the chromosomes align along a single plane, hindered the isolation of cells with highly separated chromosomes. Nevertheless, using the cytological squash technique, hundreds of individualized chromosomes could be easily distinguished in several cells. Chromosomes without visible constrictions were rod-shaped, with narrower ends (e.g., Fig. 2N–Q). Overall, *K. brevis* nuclei were larger than *K. selliformis* nuclei and much larger than *K. mikimotoi* nuclei, containing a higher number of chromosomes than the latter two species. Fig. 2 allows a comparison of the nuclear size and chromosome number of the three species.

### Physical mapping of telomeric repeats

ND-FISH using the oligonucleotide probe (CCCTAAA)_3_ to localize telomeric sequences allowed the localization of the plant-telomeric consensus sequence (TTTAGGG)n in the nuclei of the three *Karenia* species studied (Fig. 1). Hybridization signals were clearly observed at the ends of chromosomes in nuclei in which the chromosomal condensation state allowed spreading of the chromosomes (Fig. 1C–E). In the interphasic nuclei of the three *Karenia* species, intense telomeric signals were very abundantly distributed, without preferential localization in specific nuclear domains. These signals appeared in chromosomal segments that were relatively close or tethered to the nuclear envelope. Differences in the intensity of the hybridization signals were evident among the cells analyzed, but the labeling pattern was similar among the species (compare Fig. 1A and B). The stronger intensity of the abundant signals seen in interphasic nuclei than in separated chromosomes suggested a certain degree of clustering of telomeric signals during interphase. Differences in signal intensity among the well-separated chromosomes and among the telomeres of the same chromosome (Fig. 1D,E) revealed variations in telomere length. In dividing nuclei, evidenced by the presence of cytoplasmic channels crossing the nucleus (indicated in Fig. 1E) and of very condensed chromosomes, double telomeric signals were observed at the ends of some chromosomes (arrows in Fig. 1D). This occurred when the chromosomes split longitudinally in the late stages of mitosis, such that the sister chromatids became visible (arrows in Fig. 1D,E). Chromosomes with two closely located signals at the end were thicker than dividing chromatids. Hybridization signals with the telomeric probe were rarely seen at interstitial positions (arrowheads in Fig. 1D). Interestingly, the chromosomes were not randomly positioned in the nucleus during mitosis; rather, they were radially arranged, with one tip very close to the nuclear periphery and the other orientated towards the center of the nucleus (Fig. 1D,E).

### Chromosomal organization of 45S rDNA genes

Figure 2 shows the FISH results for the 45S rDNA loci *in K. brevis* (Fig. 2A–G), *K. selliformis* (Fig. 2H–J) and *K. mikimotoi* (Fig. 2K–Q). As seen by the intensity of the hybridization signals, hundreds of 45S rDNA repeats were clustered in one or multiple loci in the genomes of the studied *Karenia s*pecies, allowing the localization of nucleolar organizer regions (NORs) and nucleoli during the different cell-cycle phases. The nucleolus forms around rDNA genes during RNA transcription and ribosome biogenesis. Because DNA staining with DAPI excluded the nucleolus, the number of nucleoli was determined according to the number of less intensely DAPI-stained nuclear domains that co-localized with the 45S rDNA genes (arrow in Fig. 2E). This approach revealed enormous differences between the three species in the number of rDNA genes. Their location, beginning with the species with the largest genome size, is described below.

The nuclei of *K. brevis* contained two NORs (Fig. 2A). Based on the intensity of the signals, the presence of a similar number of repeats at one end of each of two chromosomes was inferred (Fig. 2F). In interphasic nuclei, the ribosomal clusters produced discrete double signals (Fig. 2C–E). The proximity and distance of the two rDNA loci in interphasic (Fig. 2A–E) and mitotic (Fig. 2F,G) cells, respectively, suggested the organization of a unique nucleolus throughout the cell cycle (Fig. 1E). A telophasic dividing cell is shown in Fig. 2G; the two nuclei are still present within the same cytoplasm. Note that the two NOR loci are much closer to each other in interphasic nuclei (Fig. 2A–D) than during karyokinesis (Fig. 2F,G).

A single NOR per nucleus was observed in *K. selliformis*, corresponding to the single nucleolus of this species (Fig. 2H). The 45S rDNA locus located at the termini of the single NOR-bearing chromosome is shown in Fig. 2I. Unlike in *K. brevis*, the NOR of *K. selliformis* was rarely observed as a double signal (Fig. 2J).

*K. mikimotoi* nuclei had a very high number of rDNA genes, as deduced from the number and intensity of the hybridization signals (Fig. 2K). Approximately nine NORs were observed in the nuclei with well-separated chromosomes (Fig. 2M). These nuclei contained from one to six nucleoli of variable size (Fig. 2K). The size and intensity of the hybridization signals widely differed in *K. mikimotoi* (Fig. 2L), consistent with the presence of major and minor rDNA loci located at the termini of the NOR-bearing chromosomes (Fig. 1M). Figure 2N, P shows two NOR-bearing chromosomes (from Fig. 2M, inset) that differed in their hybridization signals; the differences corresponded to the variable numbers of 45S rDNA repeats. Figure 2O, Q shows the locations of the telomeric repeats in the chromosomes, which in turn demonstrated that the rDNA genes were subtelomeric, clustered at the chromosomal termini in *Karenia*. As noted above, accurate determination of the number of NOR-bearing chromosomes in *K. mikimotoi* was not possible. Even in the well-separated chromosomal spreads obtained after FISH, the stronger signal in the confluence of two or more chromosomes could not be distinguished from fused signals from two closely positioned NOR-bearing chromosomes; hence, there may have been more than nine NOR-bearing chromosomes (Fig. 2M). The associations of NOR-bearing chromosomes in *K. mikimotoi* accounted for the variability in the number and size of the nucleoli recorded in different cells (Fig. 2K–L). Nevertheless, it was clear that, of the three studied species, *K. mikimotoi* had the highest number of NOR-bearing chromosomes and nucleoli (compare the images in Fig. 2).

### ND-FISH mapping of microsatellites

**Figure 3:**
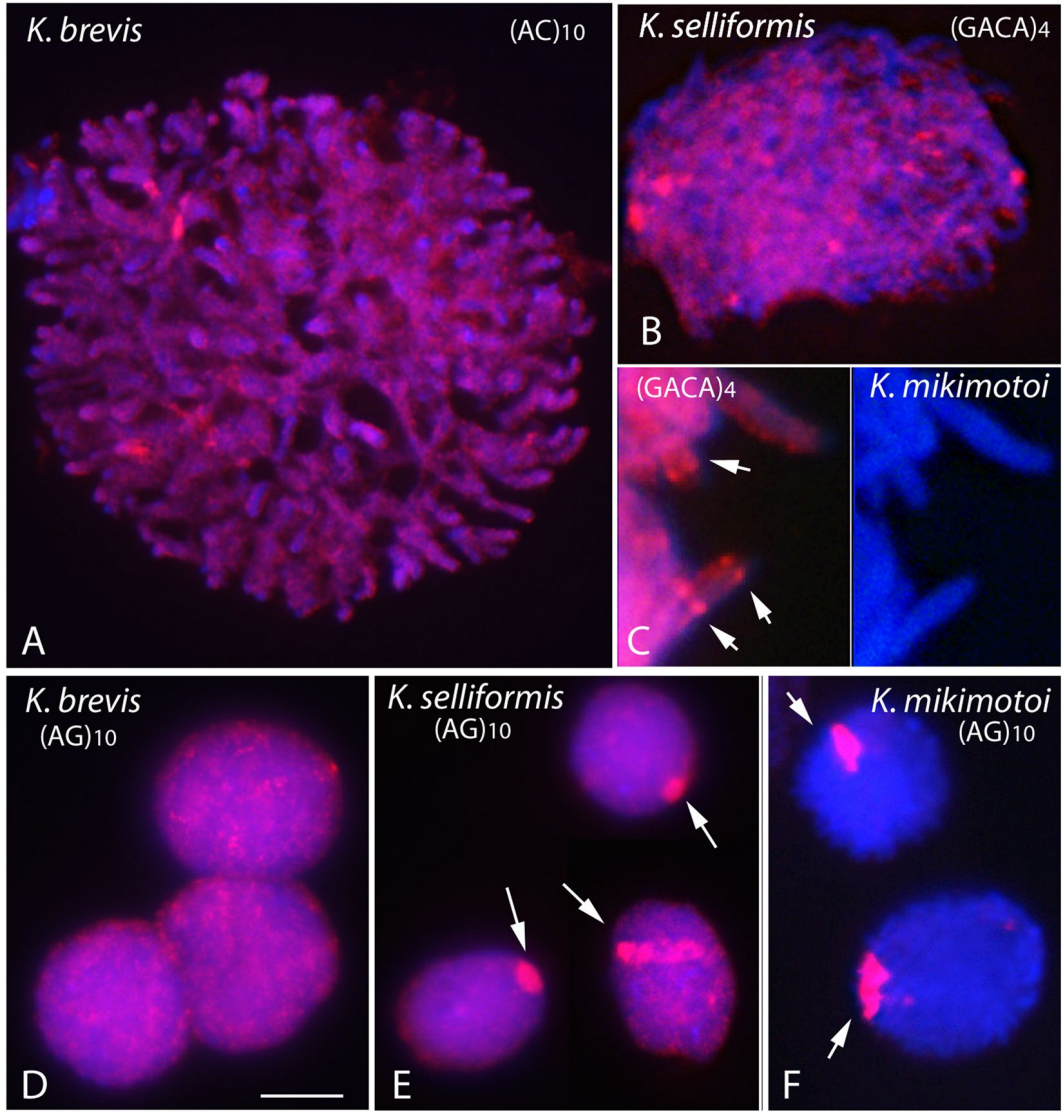
The non-random chromosomal distribution of microsatellites in three *Karenia* species. DAPI-stained DNA (blue) and *in situ* hybridization of the biotin-labeled (AC)_10_ (**A**), (GACA)_4_ (**B,C**) and (AG)_10_ (**D–F**) probes (red) in *K. brevis* (**A,D**), *K. selliformis* (**B,E**) and *K. mikimotoi* (**C,F**). A section of a nucleus with its chromosomes is shown at higher magnification in C; the arrows point to the band-like hybridization signals of different intensity. The AG-chromosome present in *K. selliformis* and *K. mikimotoi* is shown by the arrows in E and F, respectively. Note the absence of this chromosome in the nuclei of *K. brevis* cells (**D**). Scale bar = 10 μm, except in C (4 μm).

**Figure 4:**
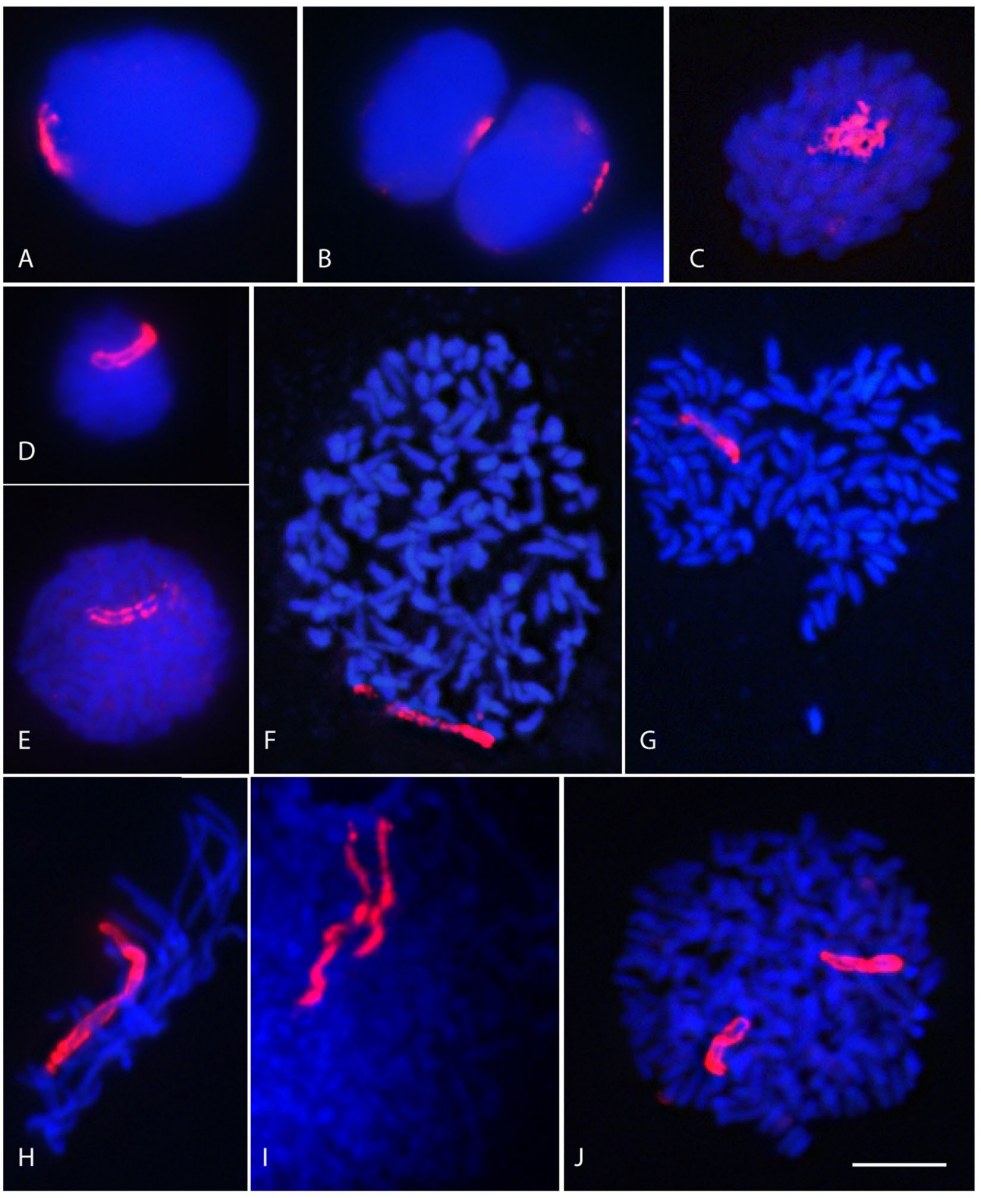
Images of the AG-chromosome during the life cycle of *Karenia mikimotoi*. DAPI-stained DNA (blue) and *in situ* detection of the biotin-labeled (AG)_10_ probe (red) in the painting of the AG-chromosome. Note the presence of a single AG-chromosome vs. a pair of AG-chromosomes in vegetative (haploids) (**A–I**) and planozygote (diploid) (**J**) cells, respectively. (**A, E**) nuclei in G2, (**B**) dividing nucleus, (**D**) a nucleus in S, (**D**) a putative gamete (**F–I**) in undergoing mitosis, (**J**) a zygote in G2. Scale bar = 10 μm.

The four microsatellite probes analyzed by ND-FISH, (AG)_10_, (AC)_10_, (GACA)_4_ and (GATA)_4_, yielded strong signals on the chromosomes of all three species (Fig. 3), indicating the widespread presence of these microsatellites in their genomes. However, the abundances of the different microsatellite motifs differed. The less intense hybridization corresponded to the GATA probe, in comparison to that of the AG, AC and GACA probes, a pattern observed in all *Karenia* species studied. The four microsatellites were generally scattered over the length of the chromosomes, with dispersed signals of variable strength interspersed between areas of lower intensity. However, clustered signals were also observed (Fig. 3A–C), occurring close together within a chromosomal region and yielding discrete band-like hybridization patterns. The distinctive bands were of different strength on the different chromosomes and were sometimes seen as two dots coinciding with sister chromatids (arrows in Fig. 3C). The densities of the AC, GACA and GATA repeats on the chromosomes of the three species did not differ, with the exception of AG. As shown in Fig. 3D–F, the density of AG repeats was higher in one chromosome of *K. selliformis* and especially in one chromosome of *K. mikimotoi*. This chromosome was referred to as the “AG-chromosome” (arrows in Fig. 3E,F). The scattered signals yielded by the AG probe that extended over the other chromosomes were masked in *K. mikimotoi* because of the intense accumulation of AG repeats in the AG-chromosome (compare Fig. 3E and F). Chromosome-specific painting probes, mostly derived from flow-sorted or the micro-manipulated dissection of chromosomes, usually contain mixtures of single-copy DNA sequences stretching over the entire length of a chromosome. However, in *K. mikimotoi*, by visualizing a single chromosome, the AG-chromosome (Fig. 4), the (AG)_10_ probe provided an efficient replacement of whole-chromosome painting.

### Visualization of the AG-chromosome during different cell-cycle and life-cycle stages in *K. mikimotoi*

The immediate advantage of (AG)_10_ when used as probe in *K. mikimotoi* cells was that it easily distinguished the presence of a single chromosome. As seen in Fig. 4, the association of the AG-chromosome with the nuclear and chromosomal morphological changes occurring during haploid asexual reproduction subsequent to the mitotic cycle (G1, S, G2 and M) could be readily followed. Fig. 4B shows the complete nuclear division that precedes the allocation of the two resulting nuclei to the two daughter cells. The image shows a high degree of symmetry in the distribution of this chromosome during division, with the maintenance of what we referred to as a distinct “I”-shaped morphology. Fig. 4C shows a nucleus with highly condensed DAPI-stained chromosomes, although the region of the nucleus occupied by the AG-chromosome maintained a spatial organization that suggested a DNA replication stage. Thus, these nuclei must correspond to the S phase of the cell cycle. Interestingly, two differentiated, more or less parallel strands of hybridization signals could be distinguished in many nuclei. This typical AG-chromosome “II”-shaped morphology (Fig. 4D) presumably corresponded to two chromatids in the replicated AG chromosomes of vegetative nuclei in the G2 stage of the cell cycle (Fig. 4E). Mitotic chromosomes (prophase-like and metaphase-like) reached a higher degree of condensation and separation (Fig. 4F–H). The two chromatids were located slightly coiled together. Accordingly, nuclei with clearly separated chromatids (Fig. 4I) were interpreted as being in an anaphase-like state, in which the separated chromatids acquired an “SS-shaped” morphology. The chromosomes differed in size, with the AG-chromosome among the longest (Fig. 4F,G).

An analysis of the dynamics of the AG-chromosome of *K. mikimotoi* during the vegetative growth of this species in culture revealed several processes related to the sexual cycle. In addition, although rarely, some nuclei contained two AG-chromosomes. As shown in Fig. 4J, in these nuclei, the overall chromosome number was twice that of nuclei with a single AG-chromosome (compare Fig. 4G and J). We interpreted these diploid cells as planozygotes. Moreover, the morphology of the two AG-chromosomes suggested replication of the respective nuclei and an association with nuclear division. The detection of zygotes in the *K. mikimotoi* cultures implied the presence of cryptic gametes among the haploid population of cells. Therefore, the rare small cells, with nuclei that were smaller than those of other cells (see Fig. 4D), were interpreted as gametes or gametocytes (compare with Fig. 4A,B or E). The presence of the two chromatids in the marker chromosome suggested that the chromosomes in the respective cells had replicated. By contrast, no cysts were observed in the cultures of *K. mikimotoi* used in the cytogenetics analysis.

## Discussion

### Genome size

Our study determined important differences in the genome sizes previously reported for the genus *Karenia*^31^. In those studies, the genome of *K. mikimotoi* was larger than that of *K. brevis* (100 pg per cell and 77.1 pg per cell, respectively), the opposite of our results for these two species. The reason for this difference is unclear but might be partially explained by the different controls and DNA fluorochromes that were used, and, in the previous study,^31^ by the fact that photosynthetic pigments were not removed nor were the cultures synchronized. An alternative explanation is the use of different strains for the analyses, as polyploidization, leading to increasing genome sizes, has been reported in some dinoflagellate strains maintained for long periods in culture^32^.

### The role of telomeres during dinochromosome division

Among the most well-known repetitive DNA sequences are those that cap the ends of linear chromosomes in eukaryotic organisms. Together with DNA-binding proteins, they form the telomeres, structures that protect chromosomes against degradation and provide a mechanism for replicating DNA at chromosome ends, among other functions. The plant telomeric consensus sequence (TTTAGGG) is present in the telomeres of the *Karenia* species analyzed in this study, and in other dinoflagellate species^23^. Dinoflagellates are eukaryotes and their chromosomes are accordingly linear, rather than circular as in prokaryotes^2^. Remarkably, their telomeric sequences form the longest tandem arrays thus far observed in unicellular organisms^24^. Telomeric sequences may also occupy interstitial positions because of translocation, inversions or other chromosomal rearrangements, but this position was only rarely seen in the chromosomes of the three *Karenia* species examined in this study.

In most eukaryotic species, the breakdown of the nuclear envelope enables chromosomal release during mitosis and the access of the spindle microtubules to the kinetochores. By contrast, in dinoflagellates, but also in other eukaryotes such as the model organism *Saccharomyces cerevisiae*, mitosis is a closed process (endomitosis), in which there is no breakdown of the nuclear envelope. In the closed mitosis of many species, a spindle assembles inside the nucleus and segregates the daughter genomes within the nuclear envelope (reviewed in^33^). However, dinoflagellates lack typical metaphase plates and kinetochores; instead, the spindle microtubules assemble outside the nucleus and access the chromosomes by attaching to the nuclear envelope through invaginations. In the closed mitosis of dinoflagellates, bundles of microtubules enclose the cytoplasmic channels adjacent to the chromosome, which remain attached to the inside of the nuclear envelope (reviewed in^34^). The chromosome ends in dinoflagellates mediate chromosomal segregation^18^^,^^23^. Electron microscopy studies have shown that dinochromosome tips are closely opposed to the nuclear envelope, to which their filaments attach, and that chromosomes display the typical V and Y configuration of dividing chromosomes, with division advancing from one end to the other^2^. Whole-mounted chromosomes examined by TEM contained a differentiated region at one chromosome end during division, consisting of two spherical conformations tightly attached to each other that could correspond to an ancient kinetochore-like structure^18^. Unlike in the mitotic chromosomes of most eukaryotes, the primary constriction denoting the presence of centromeres connecting the two sister chromatids is absent from the chromosomes of the *Karenia* species analyzed; instead, in these dinoflagellates the telomeres comprised the chromosomal region where the sister chromatids were in closest contact^18^. These results suggested that the maintenance of sister chromatid cohesion occurs at the telomeres to ensure that the chromatids stay together until anaphase and that chromosomal segregation proceeds correctly. Moreover, we observed that the telomere clusters are themselves associated with the nuclear envelope during interphase and seem to remain near the nuclear envelope during the entire cell cycle. The telomeric signals of one end were closely apposed to the nuclear envelope, which in dinoflagellate does not break down during mitosis. Thus, it may be that telomeric sequences are involved in chromosomal attachment to the nuclear envelope also during division, facilitating faithful chromosomal segregation. Interestingly, in species with holokinetic or holocentric chromosomes—in which, as in dinoflagellates, centromeric constriction is lacking and the centromere and spindle attachment sites are dispersed along the length of the chromosomes—the chromosomes may exhibit a monocentric behavior, with kinetochore activity during meiosis limited to the chromosome ends both in anaphase I and in anaphase II. For example, in some insects, kinetic activity may randomly alternate in one of the two chromosome ends (reviewed in^35^). We suggest a differential activity of opposing telomeres, similar to the function of a centromere, i.e., binding to the spindle microtubules through the nuclear envelope. Maintenance of sister chromatid cohesion could be coordinated to prevent chromosomal breakage and allow correct segregation of the chromatids to the daughter cells. Our results clearly demonstrate that *Karenia* chromosomes behave as telocentric chromosomes (i.e., with a terminal centromere). The absence of a constriction does not rule out the existence of centromeres in dinoflagellates. In most animal and plant species, the centromeres are characterized by distinct chromatin organization, epigenetics, centromere-associated proteins and histone variants. The main epigenetic marker in eukaryotes is CENP-A, a histone H3 variant that replaces histone H3 in the nucleosomes of functional centromeres. The lack of nucleosomal organization in dinoflagellates suggested that other chromatin markers direct spindle attachment and chromosomal segregation. The centromeres of higher eukaryotes are defined by hierarchical arrays of satellite repeats, including microsatellites, as shown in barley and in *Drosophila melanogaster*^36,37^. While, as far as we know, subtelomeric DNA sequences of dinoflagellates have not yet been described, it is tempting to speculate that different repetitive sequences, preferentially localized in subtelomeric regions of one of the two chromosome ends, play a role in chromosome division in dinoflagellates. None of the four microsatellite motifs analyzed in this work had a preferential subtelomeric location in the chromosomes of *Karenia*. Whether dinoflagellate chromosomes are true telocentric chromosomes, in which telomeric sequences act as canonical centromeres with kinetochore activity to drive chromosomal segregation, remains to be investigated.

### Chromosomal organization of *Karenia* 45S rDNA

Ribosomal DNA is one of the most well-characterized coding tandem arrays in eukaryotes. As in most eukaryotes, in the three *Karenia* species analyzed herein the 18S, 5,8S and 28S rRNAs genes, which are transcribed to yield the 45S ribosomal precursor and form the NOR, are clustered in discrete loci. This organization contrasts with that found in the dinoflagellate genus *Alexandrium*, in which the 45S rDNA genes are localized in specialized “ribosomal chromosomes” dedicated to the allocation of hundreds of rDNA gene copies^25^. In fact, the number of NORs differed among the three species, which demonstrated that the 45S rDNA genes can be used as chromosomal markers to discriminate between *Karenia* species. The position of the 45S rDNA sites at the termini of the chromosomes was maintained in the three species, but whether this location is genus-specific is as yet unknown. In *K. mikimotoi*, minor rDNA was detected. Minor rDNA sites have been described in several species and may be associated with nonfunctional genes or pseudogenes. The molecular identification of 28S rDNA and 18S rDNA psedudogenes in *Symbodinium*^38^ suggested that minor rDNA sites in dinoflagellates also correspond to non-functional genes. However, it is unclear whether the minor NORs of dinoflagellates are active.

The number of functional organized nucleoli may be species-specific, as little variation was observed among different cells of the same culture. In *K. brevis*, the two NORs were always associated, contributing to the formation of a single nucleolus. In *K. mikimotoi*, our results revealed the frequent fusion of two or more nucleoli, which accounted for the observed differences in the size of this vs. the other two species. In eukaryotes in general, the association of several NORs to form a single nucleolus is not uncommon, and dinoflagellates are apparently no exception, as previously reported in *Prorocentrum micans*^17^.

Significant differences in the number of repeats between species and between the 45S rDNA loci of *K. mikimotoi* were easily detectable by comparing locus sizes and the intensity of the hybridization signals. *K. mikimotoi*, the species with the lowest DNA content, had the highest number of rDNA repeats while *K. selliformis*, with an intermediate genome size, had the lowest number. Thus, in these species 45SrDNA genes did not correlate positively with genome size. As indicated above, the NOR distribution pattern observed in the three species was typically eukaryotic, which allowed *Karenia* species to be easily differentiated from species of the *Alexandrium* genus. The different NOR patterns implied important evolutionary differences. A strong positive relationship between rDNA copy number and genome size in 162 species of eukaryotes, plants and animals was previously described^39^ and was consistent with the finding of massive rDNA copy numbers in *Alexandrium*, in which the genome size is as large as 170 pg of DNA per genome^25^. The question posed in that study was whether rDNA copy number is related to the existence of the specialized chromosomes carrying them. These ribosomal chromosomes were detected in *Alexandrium* species with genome sizes > 60 pg DNA per genome. In *K. brevis*, NORs were seen only in two discrete locations although the genome size (196 ± 6.7 pg DNA per haploid genome) of this species was the largest of the three species studied and also larger than that of the *Alexandrium tamarense/catenella/fundyense* species complex (64.7 ± 7.7 pg DNA per haploid genome), in which ribosomal chromosomes have been described. Thus, our results seem to rule out a general relationship in dinoflagellates between genome size, rDNA gene copy number and the presence of ribosomal chromosomes.

### Phylogenetic relationships within *Karenia*

While *Karenia* species are in many respects morphologically similar, they differ from armoured dinoflagellates, as they are “naked”, i.e. they lack cellulosic plates inside the amphiesmal vesicles that form the rigid cell wall of thecated species. Due to the absence of a cell wall, *Karenia* species are highly pleiomorphic, resulting in a wide range of cell sizes and morphologies even within clonal cultures. This can complicate efforts to differentiate between species. Indeed, *Karenia* cells in the field cannot be easily identified using light microscopy, the method most commonly used to classify phytoplankton. Thus, non-toxic species may be misidentified as toxic species, and new species of unknown toxicity occurring during *Karenia* blooms may be overlooked. In addition, different species have been described that were later found to be the same based on DNA sequencing data, which in other cases determined that the species under investigation differed^26,40^. In the case of *Karenia*, toxic *K. brevis* is associated with Florida red tides, while *K. selliformis* and *K. mikimotoi*, first described individually in New Zealand and Japanese waters, respectively, in fact co-occur, including together with other *Karenia* species, as reported in the Gulf of Mexico^29^.

Within species, the rDNA locus number is typically stable, providing a useful marker for chromosome identification in many species. By contrast, NOR loci are highly polymorphic even between closely related taxa, and their potential intragenomic mobility is a major force that operates during speciation^41^. In a previous report we showed that NOR patterns are useful in discriminating between cryptic species within the genus *Alexandrium*^25^. Similarly, the three *Karenia* species could be easily distinguished on the basis of their 45S rDNA FISH patterns. Although a more detailed study using different strains or isolates from different geographical locations is needed to confirm this result, it is likely that the 45S rDNA distributions of different *Karenia* species are distinct and unique, perhaps reflecting their different evolutionary histories. The evolution of the chromosomal location of 45S rDNA clusters has been investigated in many different plant and animal genera. In general, the amplification of 45S rDNA and the insertion at other chromosomal locations, by transposition or other chromosomal rearrangements, of new NORs are considered to be much more likely than the removal of 45S rDNA by unequal recombination of already existing copies^42^. The phylogenetic tree inferred from the partial 28S rDNA sequence alignments clearly differentiated between *K. selliformis* and the closely related species *K. mikimotoi* and *K. brevis*^29^. The same result was inferred from phylogenetic trees based on 5.8S rDNA sequences and the internal transcribed spacers of several species and strains of the genus *Karenia*^40^. In light of this phylogenetic framework, and although the evolutionary scenario proposed herein, while the most parsimonious, needs to be confirmed in more strains and *Karenia* species, we hypothesize that the ancestral condition for the location of the *Karenia* NOR is in a single chromosome. Thus, *K. selliformis* arose first, with a single NOR-bearing chromosome, followed by the amplification of 45S rDNA sequences and the acquisition of a second NOR-bearing chromosome before the split that led to *K. brevis* and *K. mikimotoi*. The difference in the number of NORs between the latter two species would therefore have resulted from new amplifications and chromosomal rearrangements and/or transpositions of rDNA clusters, yielding multiple sites of 45S rDNA genes in *K. mikimotoi*. The increase in the number of rDNA clusters in *K. mikimotoi* was not associated with an increase in chromosome number, which rules out polyploidy and chromosome fissions as the major mechanisms of rDNA expansion in *Karenia*. Data from several genera suggest that a higher variability in NOR numbers is expected between closely related species with terminally located rDNA clusters, as is the case in *Karenia*, because they are more prone to chromosomal rearrangements such as translocation (e.g.^43,44^). Although, it is not clear which processes are responsible for rDNA amplification/dispersion or the loss of 45S rDNA, many different mechanisms may give rise to rDNA rearrangements during plant and animal species diversification. The same mechanisms may have been responsible for the changes in copy number during dinoflagellate evolution.

### Chromosomal distribution of microsatellites in *Karenia*

Microsatellite DNA, or simple/short sequences repeats (SSRs), are tandem repeat motifs of 1 to 6 base pairs found in all genomes investigated to date. As in most species, the microsatellites of dinoflagellates are highly polymorphic molecular markers that have been used to study population genetics, including of *Karenia* species^45^. A complementary approach to the extensive analysis of microsatellites by computer-based screening of DNA sequence databases constructed from genome sequencing projects is to determine the presence and distribution of microsatellites *in situ*^46^. In fact, the long-range organization of SSRs has been analyzed in a number of plants and animals species using FISH (e.g.^36,47^). In general, SSRs are scattered non-randomly throughout chromosomal DNA, between coding and non-coding regions. This can result in a specific chromosomal distribution pattern, usually associated with constitutive heterochromatin, that also serves as a chromosomal marker to distinguish specific chromosomal regions and chromosomes^48,49^.

Our study is the first to demonstrate microsatellite sites in dinoflagellate chromosomes and the universal utility of ND-FISH in the detection of microsatellite-enriched regions in eukaryotic chromosomes^47^. In agreement with data obtained in other eukaryotes, we showed the extensive accumulation of AG, AC, GACA and GATA repeats in the chromosomes of the three *Karenia* species, suggesting that chromosomal amplification and the spread of these class of tandem repeats are among the processes underlying genome evolution in *Karenia*. A correlation between microsatellite abundance and genome size in distantly related species has been proposed^50^, but the forces driving the expansion of the generally large genomes of dinoflagellates are still poorly understood. Due to these very large genomes, obtaining genomic data, especially from the highly repetitive DNA fraction, in dinoflagellates is challenging, and only annotated assembly data are available from the unusually smaller genomes (~1 Gb) of a few endosymbiont dinoflagellate species of the genus *Symbodinium*^8,9,51^. Our results suggest that microsatellites contribute substantially to the large quantity of DNA found in dinoflagellates and the important role of these repeats in DNA organization and function in *Karenia*. Indeed, in the 110-Gbp genome of *Alexandrium ostenfelddi*, >50% is made up of tandem repeats, with microsatellites accounting for 13% thereof^52^.

Microsatellites are genomic elements that change rapidly within the genome on an evolutionary timescale. By undergoing different mutation events that lead to their expansion or contraction, they are a major driver of genome evolution. Thus, the variations in AG microsatellite abundance between related *Karenia* species was not surprising. Nevertheless, the overall abundance and distribution patterns for different microsatellite repeats are generally similar between different chromosomes, with the exception of the sex chromosomes. The substantial accumulation of microsatellites during the differentiation of sex chromosomes has been documented in different organisms. For example, in birds and snakes there is a greater accumulation of GATA repeats on heterochromatic W chromosomes than on the other chromosomes of these organisms (e.g.^53^). A hallmark of the *K. selliformis* genome and especially of the *K. mikim*otoi genome is the presence of huge amounts of AG repeats on a single chromosome. Whether this AG-chromosome is involved in the same genomic function is unknown.

### Life cycle of K. *mikimotoi*

Elucidation of the life cycle of dinoflagellates is essential to an understanding of the development and dynamics of dinoflagellate blooms; however, such studies in *Karenia* are scarce and have mainly been conducted on *K. brevis*. Nevertheless, the life cycle of *K. brevis* has been characterized only partially (reviewed in^26^). As in most dinoflagellates, this species reproduces predominantly asexually, by binary fission, once every 2–10 days, with cytokinesis occurring during the day and mitosis generally at night^54^. In dinoflagellates, the sexual cycle seems to be rare, occurring only under stressful environmental conditions and often species-specific. However, recent studies have shown that the sexual cycle is probably much more frequent and plastic than previously estimated (see review in^20^). Morphological studies of different *Karenia* species in culture have identified morphologies compatible with a sexual cycle, including spherical cyst-like cells, although a sexual origin of these cells has never been confirmed. In addition, pairs of isogamous gametes have been observed in cultures of *K. mikimotoi*, albeit with a low incidence^55^. The mixing of different clones of *K. brevis* in culture results in the higher occurrence, aggregation and fusion of isogamous gametes, as well as the formation of planozygotes^28^. Thus, homothallic (gametes of the same mating type) and heterothallic behaviors may be simultaneously present depending on the strain.^56^ Gametes and planozygotes are morphologically similar to vegetative cells, but gametes tend to be smaller and rounder while planozygotes are larger and bear two longitudinal flagella instead of the single longitudinal flagellum characteristic of the vegetative stage. By contrast, hypnozygotes or resting cysts have not yet been reported in *Karenia*.

**Figure 5:**
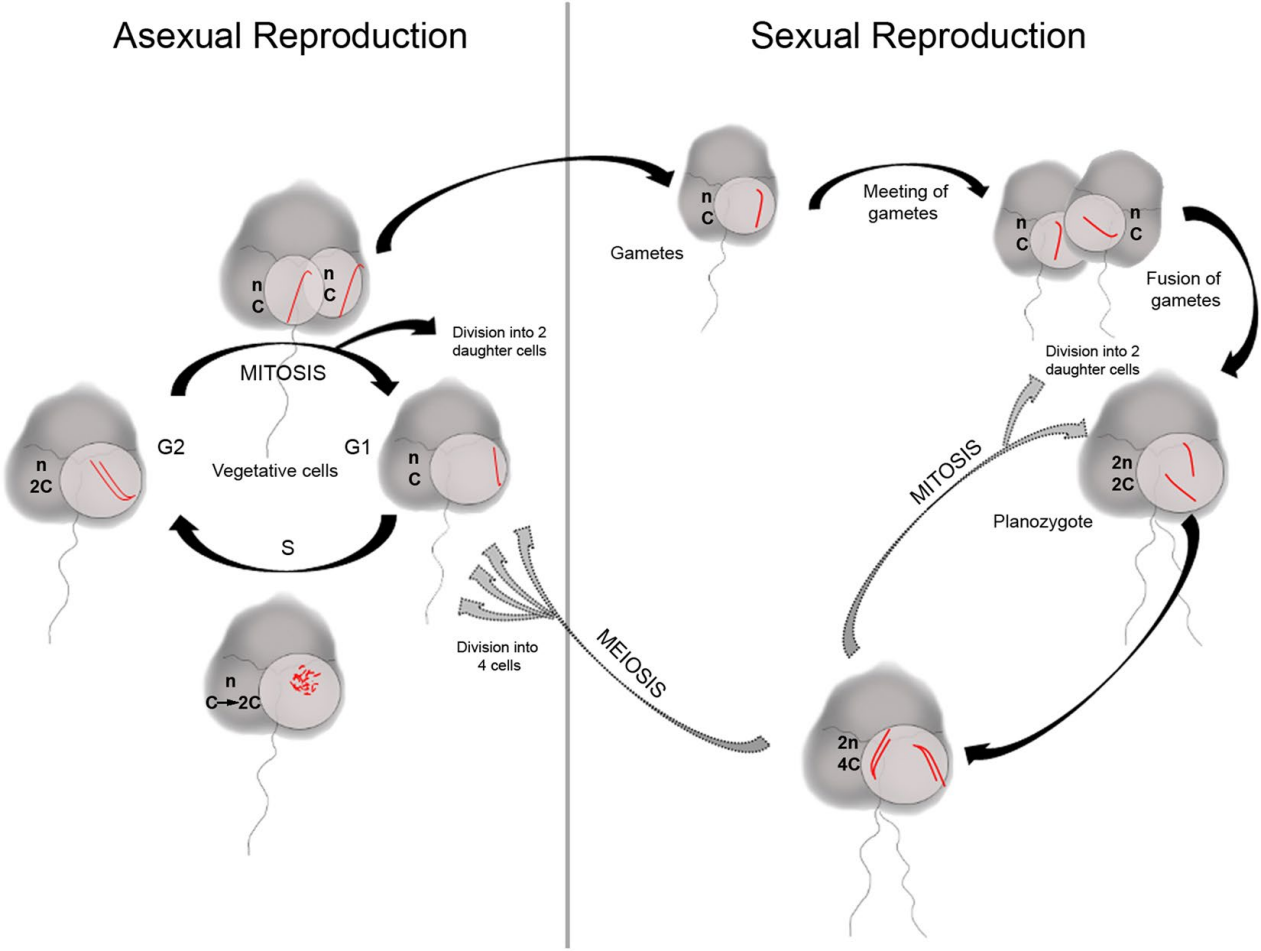
Schematic diagram of the processes that may take place during the asexual and sexual phases of the genus *Karenia*. A single chromosome is drawn in red to follow its changes during the dinoflagellate life cycle. Note that planozygotes may undergo meiotic and mitotic divisions.

This study is the first to follow a single chromosome in dinoflagellates in order to monitor the changes in nuclear morphology that occur during the proliferative asexual phase. Figure 5 is a schematic diagram of the processes proposed to take place in *K. mikimotoi* cells during the various cell-cycle stages. The ability to visualize one or two of the chromatids in the AG-chromosome enabled us to distinguish haploid (n) vegetative cells during asexual reproduction, with a DNA content of C in G1, before cell replication in S, leading to cells with a 2 C DNA content during G2 and mitosis after replication. In *K. mikimotoi*, at least in the strain studied herein, our data support the existence of sexual reproduction, although with a low incidence, based on the detection of diploid cells (2n). Moreover, our data provide evidence of planozygote division. A marker of zygote division is the presence of 4 C cells, which occur only in diploid (zygote) cells (2 C) after replication. But what is the nature of planozygote division? In dinoflagellates species that do not form resting cysts, planozygotes are thought to divide by meiosis, which restores the haploid content of vegetative cells. However, planozygotes may enter routes of division other than diploid-haploid turnover. For example, in a previous study we showed that the planozygotes of *Alexandrium minutum* divide also via mitosis^57^. Other authors have proposed that planozygotes in species such as *Polykrikos kofoidii* divide by meiosis and mitosis^58^. A biphasic cycle has also been described in the yeast *Schizosaccharomyces pombe*, the zygotes of which undergo meiosis or mitosis depending on nutritional factors^59^. Although in this study we did not further investigate planozygote division, new insights could be obtained in future studies by monitoring the AG-chromosome in crosses of different *K. mikimotoi* strains under conditions aimed at increasing planozygote formation. Whether *Karenia* species have a haplodiplontic life cycle, with individuals undergoing mitotic division in both the sexual (diploid) and asexual (haploid) phases, could be easily revealed by monitoring the AG-chromosome in sexually induced crossed cultures. As illustrated in Fig. 5, we were able to discriminate among haploid (n) cells with a DNA content of C or 2 C (before or after replication) and diploid (2n) cells with a G1 and G2 DNA content of 2 C and 4 C, respectively, by following the dynamics of the AG-chromosome. Diploid cells at 4 C stages will disappear if division is via meiosis, with a shift to haploid vegetative cells. In this case, and if the diploid planozygotes undergo meiosis, there will be a transition from 2 C (G1) to 4 C (G2) after replication, before division finally yields haploid cells with a C DNA content in G1. Alternatively, an increase in the number of diploid cells with a DNA content of 2 C (G1) or 4 C (G2), depending the replication state of the cells, will reveal whether planozygotes divide by mitosis. These two scenarios, proposed as possible life-cycle modes of planozygote division, are depicted in Fig. 5.

Nuclear cyclosis in diploid stages and associated with meiosis has been observed In some species (e.g.^60^). However, the phases of meiosis in dinoflagellates have not been characterized in any detail nor have chromosomal recombination and segregation been documented. Nevertheless, inheritance studies support the occurrence of genetic recombination and thus meiosis^61,62^. One of the reasons for this lack of knowledge on dinoflagellate meiosis is that chromosome segregation patterns have not been described in these organisms, probably due to their large number of chromosomes. This problem can be overcome by analyzing the behavior during meiosis of only one pair of homologous chromosomes in the zygotes, as demonstrated herein using the AG-chromosome. Some studies have indicated that dinoflagellate meiosis is an unusual one-division process^63^, which would imply that the zygote DNA content does not undergo a 2 C to 4 C transition, as there is no replication of chromatids. However, our results convincingly show that zygote replication takes place. If the division of planozygotes by mitosis is excluded, then, at least in *K. mikimotoi*, a typical two-step meiosis after DNA replication becomes plausible.

In many dinoflagellates, including *Karenia*, gametes (mating cells) are morphologically similar but slightly smaller than vegetative cells. Interestingly, the smallest *K. brevis* cells previously observed in culture^21^, in a study of their sexual life stages at different temperatures, did not seem to mate, suggesting the necessity of two consecutive divisions for gamete formation in this species, as also reported for *Gyrodinium uncatenum*^64^. In our study, replication of the DNA of cells with the smallest nuclei provides support for a round of division before gamete differentiation.

## Conclusions

The use of different FISH methods, including the simple ND-FISH assay, to develop physical markers in *Karenia* provides obvious advantages in investigations of the organization and behavior of the chromosomes of these dinoflagellates during their different cell-cycle and life-cycle stages. In future studies, the developed markers can be combined with flow cytometry analyses in order to achieve a more accurate identification of cell and life cycle stages. Moreover, this approach can be applied to other dinoflagellate species and may enable a better characterization of the biology of their complex chromosomes. Telomeres are far more than the protective ends of chromosomes and the complete spectrum of their numerous other functions has yet to be elucidated. However, our study suggests the intimate involvement of telomeres in ensuring proper chromosomal segregation in dinoflagellates during mitosis, as these chromosomes, without known centromeres, exhibit a telocentric behavior. We also identified several chromosomal attributes, such as sister chromatids, and thus the greater than expected resemblance of dinoflagellate chromosomes to the chromosomes of other eukaryotes. Our results showed that the species analyzed herein can be easily distinguished on the basis of their 45S rDNA and AG hybridization patterns, but an understanding of the origin of the remarkable differences in the amount and location of both the 45S rDNA genes and AG repeats between *Karenia* species awaits further studies, as do determinations of the relationship between *Karenia* and other dinoflagellate genera and whether the evolutionary trends of the studied species are consistent with the known phylogeny. Also unclear is whether the spreading of other microsatellite elements occurs in the genus *Karenia* and the origin and function of the AG-chromosome. Although it is widely assumed that sex is rare in dinoflagellates, in this study we were able to show the existence of sexual life stages in non-induced cultures. The presence of the AG-chromosome and its easy detection are convenient features of the *K. mikimotoi* genome that can be used in future studies of sexuality in *Karenia*, an aspect of its biology that, as in other dinoflagellates, is thought to underlie recurrences of their blooms.

## Methods

The strains of *K. mikimotoi*, KT77B, *K. selliformis* GM94GAB and *K. brevis* CCMP2281 employed in this study (Table 1) are regularly maintained at the Centro Oceanográfico de Vigo (CCVIEO; the Culture Collection of Harmful Microalgae of the Spanish Institute of Oceanography).

**Table 1:** List of strains employed and estimated genome size (pg DNA, present study).

Strain	Species	Location of isolation	Origin/Clonal	Collection year	Genome size
CCMP2281	*Karenia brevis* ^70– 72^	Gulf of México, USA	Bloom/Yes	1999	196.7 ± 6.7
KT77B	*Karenia mikimotoi* ^72, 73^	Oslosfjorden, Norway	Unknown/No	1977	53.4.1 ± 3.05
GM94GAB	*Karenia selliformis* ^74^	Gulf of Gabes, Tunesia	Bloom/No	1994	158.6 ± 9.5

### Culture conditions

The strains were cultured at 20 °C with an irradiance of ~90 μmol photons m^−2^s^−1^ and a photoperiod of 12:12 h L:D (light:dark). Culture stocks were maintained in Iwaki 50-mL flasks filled with 30 mL of L1 medium^65^ without added silica. The medium was prepared using Atlantic seawater adjusted to a salinity of 30 psu by the addition of sterile distilled water.

### Flow cytometry

Exponentially growing cultures were incubated for 48 h in the dark to synchronize cell division^66,67^. Fifty mL of culture were filtered through a 5.0-μm pore size membrane filter (Millipore, Ireland), fixed with 1% paraformaldehyde for 10 min, washed in PBS (pH 7, Sigma-Aldrich, St. Louis, USA) and then centrifuged at 1200 g × 10 min. The pellet was resuspended in 2 mL of cold methanol and stored for at least 12 h at 4 °C to achieve chlorophyll extraction. The cells were then washed twice in PBS and the pellet was resuspended in 300 μL of propidium iodide (60 μg mL^−1^) and 30 μL of RNaseA (100 mg mL^−1^) for at least 2 h before analysis using a Sony SH800Z flow cytometer with a laser emitting at 488 nm. Duplicate samples were run at low speed and data were acquired in linear and log modes until at least 10000 events had been recorded. After aggregates and dividing cells (2 C) were discarded, gated *Karenia* vegetative populations (1 C) accounted for > 8000 events. The size of the smallest genome (*K. mikimotoi*) was calculated based on the size of the genomes of *Alexandrium minutum* strains VGO577 and AMP4^68^. The fluorescence emission of propidium iodide was detected at 620 nm. FCS Express 6 (De Novo Software, USA) was used to compute peak numbers, coefficients of variation (CVs) and peak ratios for the DNA fluorescence distributions in a population.

### Slide preparation

Saturated cell suspensions of unsynchronized cultures were used in the cytogenetic analysis. Although the culture could to some extent be synchronized naturally by the light:dark period, different cell-cycle stages within the culture were expected. The cells were harvested by gentle centrifugation (at 1500 g, 5 min) to remove the L1 medium and the pellet was resuspended in a final volume of 5 mL of 0.2 μl colcemid (Gibco Life Technologies, UK)/ml for 3.5 h to obtain a high proportion of mitotic cells. The cells were then pelleted and the pellet resuspended in Carnoy’s solution (ethanol:acetic acid, 3:1, v/v), followed by fixation for at least 24 h. The samples were prepared using the squash method. Fixed cells were harvested by centrifugation (7000 rpm, 10 min) and the pellet, containing thousands of cells, resuspended in a drop of 45% acetic acid before a drop of the suspension was placed on a microscopy slide. The slides were heated briefly over a flame to remove the cytoplasm. After removal of the cover slips by freezing the slides, the samples were air-dried.

### DNA probes and labeling

The DNA probe used for mapping the rDNA genes was pTa71. This plasmid contains a 9-kb *Eco*RI fragment from *Triticum aestivum* that includes the 45S rDNA region^69^. pTa71 was labeled with digoxigenin-11-dUTP using a kit from Sigma-Aldrich (Dig-nick translation mix).

Telomeric repeats at both ends were detected using the single-strand oligonucleotide (CCCTAAA)_3_, synthesized with Dy547 (red) (Isogen Life Science).

Four oligonucleotides, (AG)_10_, (AC)_10_, (GATA)_4_ and (GACA)_4_, supplied with biotin incorporated at both ends (Isogen Life Science), were used to analyze the microsatellites. They are representative of the most abundant microsatellite motifs clustered at the chromosomal level in the species so far analyzed^47^.

### Non-denaturing (ND)-FISH

The slides were directly incubated at room temperature (RT) in a humidity chamber for 2 h with 30 μL of hybridization mixture containing 2 pmol of the oligonucleotide probe in 2 × SSC. For post-washing, the slides were immersed in 4 × SSC/0.2% Tween20 and agitated for 10 min at RT. The probes were detected as described above.

### FISH

The slides were incubated with RNase A, fixed in 4% (w/v) paraformaldehyde, dehydrated in a graded ethanol series and air-dried as previously described^23^. Thirty μL of hybridization mixture (50% deionized formamide, 10% dextran sulfate, 2 × SSC, and 0.33% SDS) containing 100 ng of the digoxigenated pTa71 probe was applied to each slide after denaturation of the probe in an oven for 10 min at 70 °C. When two-color FISH was carried out, in combination with the oligonucleotide probes, to detect pTa71, 2 pmol of the chosen oligonucleotide was included in the hybridization mixture. Denaturation was achieved by placing the slides in an incubator at 75 °C for 7 min, with the temperature controlled using a programmable thermal controller (PT-100, M.J. Research Inc.). Hybridization was performed in a humidified chamber by incubating the slides overnight at 37 °C. Non-specific signals were removed by washing the slides in Coplin jars with 4 × SSC/Tween^20^, with agitation, for 10 min at RT before specific signal detection.

### Probe detection, microscopy and imaging

The bound digoxigenin and biotin probes were detected by incubating the slides in fluoresceinated anti-digoxigenin (Roche Applied Science) and streptavidin-Cy3 (Sigma-Aldrich), respectively, prepared in 5% (w/v) BSA, for 1 h at 37 °C. No immunocytochemical procedures were required for the detection of the Dy-547-labeled telomeric probe. Before staining the DNA with DAPI (4′, 6-diamidino-2-phenylindole), the slides were rinsed for 10 min in 4 × SSC/Tween^20^ at RT. Finally, the slides were mounted in antifade solution (Vector Laboratories). Hundreds of nuclei were analyzed using an epifluorescence Axiophot Zeiss system. Images were captured with a cooled CCD camera (Zeiss) and merged using Adobe Photoshop. The images were optimized for best contrast and brightness using the same program but only those functions that treated all pixels in the image equally.

## Electronic supplementary material

Supplementary information

## Data Availability

The dinoflagellate strains used in this study belong to the public collection of the Spanish Institute of Oceanography in Vigo (Spain). All data generated or analyzed during this study are included in this published article (and its supplementary information files).

## References

[1] Hoppenrath, M., Murray, S. A., Chomérat, N. & Horiguchi, T. Marine benthic dinoflagellates-unveiling their world-wide biodiversity. *Kleine Senchenber Reihe* **54** (2014).

[2] Soyer-Gobillard MO, Geraud ML (2015). Chromosomes of protists: The crucible of evolution. Int. Microbiol..

[3] Moreno Díaz de la espina S, Alverca E, Cuadrado A, Franca S (2005). Organization of the genome and gene expression in a nuclear envionment lacking histones and nucleosomes:the amazingdinoflagellates. Eur. J. Cell Biol..

[4] Wisecaver H, Hackett JD (2011). Dinoflagellate genome evolution. Annu. Rev. Microbiol..

[5] Murray SA (2016). Unravelling the functional genetics of dinoflagellates: a review of approaches and opportunities. Perspect. Phycol..

[6] Lin S (2011). Genomic understanding of dinoflagellates. Res.Microbiol..

[7] Roy S, Morse D (2012). A full suite of histone and histone modifying genes are transcribed in the dinoflagellate Lingulodinium. PLoS One.

[8] Shoguchi E (2013). Draft assembly of the Symbiodinium minutum nuclear genome reveals dinoflagellate gene structure. Curr. Biol..

[9] Lin S (2015). The symbiodinium kawagutti genome illuminates dinoflagellate gene expression and coral symbiosis. Science (80-.)..

[10] Hackett JS, Anderson DM, Erdner D, Bhattacharya D (2004). Dinoflagellates: a remarkable evolutionary experiment. Am. J. Bot.

[11] Zhang H (2007). Spliced leader RNA trans-splicing in dinoflagellates. Sci., Proc. Natl. Acad..

[12] Chow MH, Yan KTH, Bennett MJ, Wong JTY (2010). Birefringence and DNA condensation of liquid crystalline chromosomes. Eukaryot. Cell.

[13] Gornik SG (2012). Loss of nucleosomal DNa condensation coincides with appearance of a novel nuclear protein in dinoflagellates. Curr. Biol..

[14] Shi, X., Ma., M., Lin, S. Cell cycle-dependent expression dynamics of G1/S specific cyclin, cellulose synthase and cellulose in the dinoflagellate Prorocentrum donghaiense. *Front. Microbiol*. **8** (2017). 10.3389/fmicb.2017.01118 PMC5476699 28676796

[15] Dapena C, Bravo I, Cuadrado A, Figueroa RI (2015). Nuclear and Cell Morphological Changes during the Cell Cycle and Growth of the Toxic Dinoflagellate Alexandrium minutum. Protist.

[16] Bhaud Y (2000). Morphology and behaviour of dinoflagellate chromosomes during the cell cycle and mitosis. J.Cell Sci..

[17] Soyer-Gobillard MO, Geraud ML (1992). Nucleolus behavior during the cell cycle of a primitive dinoflagellate eukaryote, Prorocentrum micans Her., seen by light microscopy and electron microscopy. J. Cell Sci..

[18] Costas E, Goyanes VJ (2005). Architecture and evolution of dinoflagellate chromosomes. An enigmatic origin. Cytogenet. Genome Reserach.

[19] von Dassow P, Montresor M (2011). Unveiling the mysteries of phytoplankton life cycles: patterns and opportunities behind complexity. J. Plankton Res..

[20] Figueroa RI, Estrada M, Garcés E (2018). Life histories of microalgal species causing harmful blooms: Haploids, diploids and the relevance of benthic stages. Harmful Algae.

[21] Persson A, Smith BC, Wikfors GH, Alix JH (2013). Differences in swimming pattern between life cycle stages of the toxic dinoflagellate Alexandrium fundyense. Harmful Algae.

[22] Moreau H, Geraud ML, Bhaud Y, Soyer-Gobillard M (1998). O. cloning, characterization and chromosomal localization of a repeated sequence in Crypthecodinium cohnii, a marine dinoflagellate. Int. Microbiol..

[23] Alverca E, Cuadrado A, Jouvé N, Franca S (2007). Moreno Díaz de la Espina, S. Telomeric DNA localization on dinoflagellate chromosomes: structural and evolutionary implications. Cytogenet. Genome Res..

[24] Fojtová M (2010). Telomere maintenance in liquid crystalline chromosomes of dinoflagellates. Chromosoma.

[25] Figueroa, R. I., Cuadrado, A., Stüken, A., Rodríguez, F. & Fraga, S. Ribosomal DNA Organization Patterns within the Dinoflagellate Genus Alexandrium as Revealed by FISH: Life Cycle and Evolutionary Implications. *Protist* **165** (2014). 10.1016/j.protis.2014.04.001 24846057

[26] Brand LE, Campbell L, Bresnan E (2012). Karenia: the biology and ecology of a toxic genus. Harmful Algae.

[27] da Silva PM (2008). Immunological responses of the Manila clam (Ruditapes philippinarum) with varying parasite (Perkinsus olseni) burden, during a long-term exposure to the harmful alga, Karenia selliformis, and possible interactions. Toxicon.

[28] Walker LM (1982). Evidence for a Sexual Cycle in the Florida Red Tide Dinoflagellate, Ptychodiscus brevis (=Gymnodinium breve). Trans. Am. Microsc. Soc..

[29] Haywood AJ (2004). Comparative morphology and molecular phylogenetic analysis of three new species of the genus Karenia (Dinophyceae) from New Zealand. J. Phycol..

[30] Miguel F. de Salas C, Bolch JS, Hallegraeff GM (2004). Karenia umbella sp. nov. (Gymnodiniales, Dinophyceae), a new potentially ichthyotoxic dinoflagellate species from Tasmania, Australia. Phycologia.

[31] LaJeunesse TC, Lambert G, Andersen RA, Coffroth MA, Galbraith DW (2005). Symbiodinium (Pyrrhophyta) genome sizes (DNA content) are smallest among dinoflagellates. J. Phycol..

[32] Loper CL, Steidinger KA, Walker LM (1980). A simple chromosome spreadtechnique for unarmoured dinoflagellates and implications of polyploidy in algal cultures. Trans. Am. Microsc. Soc..

[33] Sazer S, Lynch M, Needleman D (2014). Deciphering the evolutionary history of open and closed mitosis. Curr. Biol..

[34] Drechsler H, McAinsh AD (2012). Exotic mitotic mechanisms. Open Biol..

[35] Guerra M (2010). Neocentrics and holokinetics (holocentrics): chromosomes out of the centromeric rules. Cytogenet. Genome Res..

[36] Cuadrado A, Jouve N (2007). Similarities in the chromosomal distribution of AG and AC repeats within and between Drosophila, human and barley chromosomes. Genome Res..

[37] Cuadrado, A., Jouve, N. Novel simple sequence repeats (SSRs) detected by ND-FISH in heterochromatin of Drosophila melanogaster. *BMC Genomics* **12** (2011). 10.1186/1471-2164-12-205 PMC3114746 21521504

[38] Santos SR, Sakai K, Kinzie RA, Coffroth MA (2003). Molecular Characterization of Nuclear Small Subunit (18S)-rDNA Pseudogenes in a Symbiotic Dinoflagellate (Symbiodinium, Dinophyta). J. Eukaryot. Microbiol..

[39] Prokopowich CD, Gregory TR, Crease TJ (2003). The correlation between rDNA copy number and genome size in eukaryotes. Genome.

[40] Yamaguchi H (2016). Occurrence of Karenia papilionacea (Dinophyceae) and its novel sister phylotype in Japanese coastal waters. Harmful Algae.

[41] Schubert I (2007). Chromosome evolution. Curr. Opin. Plant Biol..

[42] Hasterok R (2006). Comparative analysis of rDNA distribution in chromosomes of various species of Brassicaceae. Ann. bot..

[43] Pedrosa-Harand A (2006). Extensive ribosomal DNA amplification during Andean common bean (Phaseolus vulgaris L.) evolution. Theor. Appl. Genet..

[44] Nguyen P, Sahara K, Yoshido A, Marec F (2010). Evolutionary dynamics of rDNA clusters on chromosomes of moths and butterflies (Lepidoptera). Genetica.

[45] Nagai S, Yasuike M, Nakamura Y, Tahvanainen P, Kremp A (2015). Development of ten microsatellite markers for Alexandrium ostenfeldii, a bloom-forming dinoflagellate producing diverse phycotoxins. J. Appl.Phycol..

[46] Ruiz-Ruano FJ, Cuadrado A, Montiel EE, Camacho JP, López-León MD (2015). Next generation sequencing and FISH reveal uneven and nonrandom microsatellite distribution in two grasshopper genomes. Chromosoma.

[47] Cuadrado A, Jouve N (2010). Chromosomal detection of simple sequence repeats (SSRs) using nondenaturing FISH (ND-FISH). Chromosoma.

[48] Cuadrado A, Schwarzacher T (1998). The chromosomal organization of simple sequence repeats in wheat and rye genomes. Chromosoma.

[49] Cuadrado A, Cardoso M, Jouve N (2008). Physical organization of simple sequence repeats (SSRs) in Triticeae: structural, functional and evolutionary implications. Cytogenet. Genome Reserach.

[50] López-Flores I, Garrido-Ramos MA (2012). The repetitive DNA content of eukaryotic genomes. Genome Dyn..

[51] M. Aranda *et al*. Genomes of coral dinoflagellate symbionts highlight evolutionary adaptations conducive to a symbiotic lifestyle. *Sci. Rep*. **6** (2016). 10.1038/srep39734 PMC5177918 28004835

[52] Jaeckisch N (2011). Comparative Genomic and Transcriptomic Characterization of the Toxigenic Marine Dinoflagellate Alexandrium ostenfeldii. PLoS One.

[53] O’Meally D (2010). Non-homologous sex chromosomes of birds and snakes share repetitive sequences. Chromosom. Res..

[54] Van Dolah FM (2009). K. The Florida red tide dinoflagellate Karenia brevis: New insights into cellular and molecular processes underlying bloom dynamics. Harmful Algae.

[55] Ouchi A, Aida S, Uchida T, Honjo T (1994). Sexual reproduction of a red tide dinoflagellate Gymnodinium mikimotoi. Fish. Sci..

[56] Steidinger, K. A., Vargo, G., Tester, P. & Tomas, C. In *Physiological Ecology of Harmful Algal Blooms* 133–153 (1998).

[57] Figueroa RI, Dapena C, Bravo I, Cuadrado A (2015). The hidden sexuality of Alexandrium minutum: An example of overlooked sex in dinoflagellates. PLoS One.

[58] Tillmann U, Hoppenrath M (2013). Life Cycle of the pseudocolonial dinoflagellate Polykrikos kofoidii (Gymnodiniales, Dinoflagellata). J. Phycol..

[59] Davis L, Smith GR (2001). Meiotic recombination and chromosome segregation in Schizosaccharomyces pombe. Proc. Natl. Acad. United States Am..

[60] Gribble KE, Anderson DM, Coats DW (2009). Sexual and asexual processes in Protoperidinium steidingerae Balech (Dinophyceae), with observations on life-history stages of Protoperidinium depressum (Bailey) Balech (Dinophyceae). J. Eukaryot. Microbiol..

[61] Figueroa, R. I., Rengefors, K. & Bravo, I. Effects of parental factors and meiosis on sexual offspring of Gymnodinium nolleri (Dinophyceae). *J. Phycol*. **42** (2006).

[62] Figueroa, R. I., Garcés, E. & Bravo, I. Comparative study of the life cycles of Alexandrium tamutum and Alexandrium minutum (Gonyaulacales, Dinophyceae) in culture. *J. Phycol*. **43** (2007).

[63] Beam CA, Himes M, Himelfarb J, Link C, Shaw K (1977). Genetic evidence of unusual meiosis in the dinoflagellate Crypthecodinium cohnii. Genetics.

[64] Coats DW, Tyler MA, Anderson DM (1984). Sexual processes in the life cycle of Gyrodinium uncatenum (Dinophyceae): A morphogenetic overview. J. Phycol..

[65] Guillard RRL, Hargraves PE (1993). Stichochrysis immobilis is a diatom, not a chrysophyte. Phycologia.

[66] Figueroa, R. I., Garcés, E. & Bravo, I. The use of flow cytometry for species identification and life-cycle studies in dinoflagellates. *Deep. Res. Part II Top. Stud. Oceanogr*. **57** (2010).

[67] Taroncher-Oldenburg G, Kulis DM, Anderson DM (1997). Toxin variability during the cell cycle of the dinoflagellate Alexandrium fundyense. Limnol. Oceanogr..

[68] Stüken, A. *et al*. Paralytic shellfish toxin content is related to genomic sxtA4 copy number in Alexandrium minutum strains. *Front. Microbiol*. **6** (2015). 10.3389/fmicb.2015.00404 PMC4416454 25983733

[69] Gerlach WL, Redbrook JR (1979). Cloning and characterization of ribosomal RNA genes from wheat and barley. Nucleic Acids Res..

[70] Daugbjerg N, Hansen G, Larsen J (2000). M. Ø. Phylogeny of some of the major genera of dinoflagellates based on ultrastructure and partial LSU rDNA sequence data, including the erection of three new genera of unarmoured dinoflagellates. Phycologia.

[71] Snyder RV (2005). Localization of polyketide synthase encoding genes to the toxic dinoflagellate Karenia brevis. Phytochemistry.

[72] Al-Kandari MA, Highfield AC, Hall MJ, Hayes P, Schroeder DC (2011). Molecular tools separate harmful algal bloom species, Karenia mikimotoi, from different geographical regions into distinct sub-groups. Harmful Algae.

[73] Hansen G, Daugbjerg N, Henriksen P (2000). Comparative study of Gymnodinium mikimotoi and Gymnodinium aureolum, comb. nov. (=Gyrodinium aureolum) based on morphology, pigment composition, and molecular data. J. Phycol..

[74] Hansen, G., Erard Le Denn, E., Daugbjerg, N. & Rodríguez, F. *Karenia selliformis responsible for the fish-kills in the Gulf of Gabes, Tunisia 1994*. IFREMER (2004).

