# Supplementary material for: Chromosomal markers in the genus Karenia: Towards an understanding of the evolution of the chromosomes, life cycle patterns and phylogenetic relationships in dinoflagellates
Source: Sci Rep. 2019 Feb 28;9:3072. doi: 10.1038/s41598-018-35785-7 (PMC6395649; doi:10.1038/s41598-018-35785-7)
Supplement: Supplementary file 1 — Supplementary information [file 41598_2018_35785_MOESM1_ESM.pdf]

SUPPLEMENTARY INFORMATION:

Chromosomal markers in the genus *Karenia*: Towards an understanding of the evolution of the chromosomes, life cycle patterns and phylogenetic relationships in dinoflagellates

Ángeles Cuadrado[a,1], Alfredo de Bustos[a], Rosa I. Figueroa[b,c,1]

[a] Universidad de Alcala (UAH), Dpto Biomedicina y Biotecnología, 28805 Alcalá de Henares, Madrid, Spain
[b] Instituto Español de Oceanografia (IOE), Subida a Radio Faro 50, 36390 Vigo, Spain
[c] Aquatic Ecology, Biology Building, Lund University, 22362 Lund, Sweden

[1]Corresponding authors
Angeles Cuadrado e-mail: angeles.cuadrado@uah.es
Rosa I. Figueroa e-mail: rosa.figueroa@ieo.es

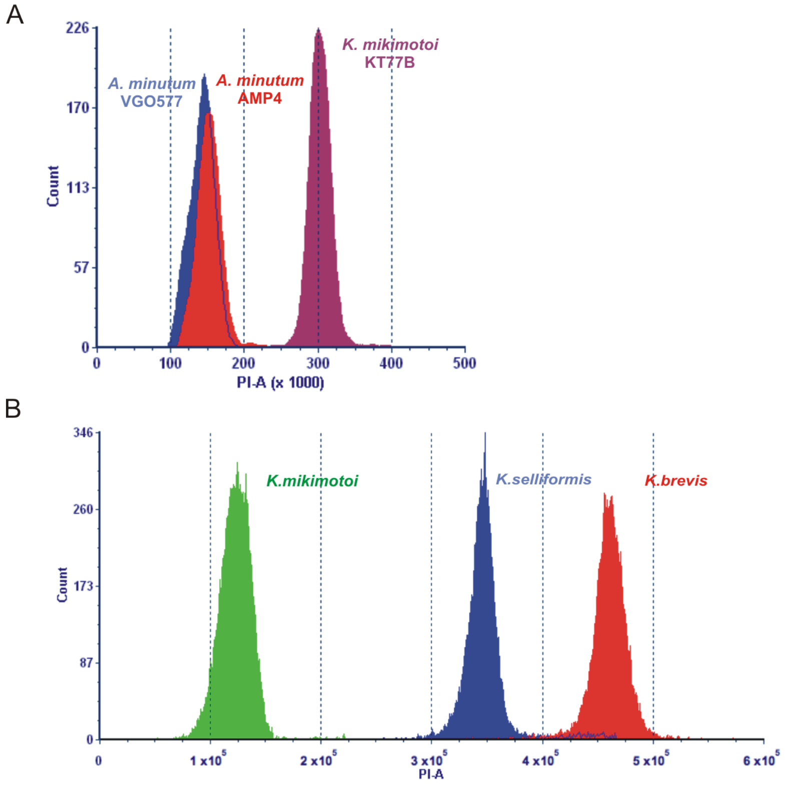

Fig. S1. **Genome size estimations by flow cytometry. A.** Relative estimation of the size of the *Karenia mikimotoi* genome in relation to the genome sizes of two *Alexandrium minutum* controls. **B.** Comparisons of the genome sizes of the three *Karenia* species. PI-A: propidium iodide fluorescence at a linear scale (area). Single peaks correspond to the G1 (1C) stage of the cell cycle.
